# Retraction: Bridging the mental health gap: unveiling and mitigating the hidden toll of workplace behaviors on diverse populations

**DOI:** 10.3389/fpubh.2024.1508050

**Published:** 2024-10-15

**Authors:** Frontiers Editorial Office

The journal retracts the 17 November 2023 article cited above.

Following publication, concerns were raised regarding the scientific validity of the article. An investigation was conducted in accordance with Frontiers' policies.

It was found that the concerns were valid and that the article does not meet the standards of editorial and scientific soundness for Frontiers in Public Health; therefore, the article has been retracted.

The retraction was approved by the Chief Editors for Frontiers in Public Health and the Chief Executive Editor of Frontiers. The author agrees to this retraction.

